# Awareness of age-related change in very different cultural-political contexts: A cross-cultural examination of aging in Burkina Faso and Germany

**DOI:** 10.3389/fpsyt.2022.928564

**Published:** 2023-01-20

**Authors:** Anton Schönstein, Anna Schlomann, Hans-Werner Wahl, Till Bärnighausen

**Affiliations:** ^1^Network Aging Research, Heidelberg University, Heidelberg, Germany; ^2^Department of Psychology, Mannheim University, Mannheim, Germany; ^3^Institute for Educational Sciences, Heidelberg University of Education, Heidelberg, Germany; ^4^Institute of Psychology, Heidelberg University, Heidelberg, Germany; ^5^Heidelberg University, Heidelberg, Germany

**Keywords:** views on aging, AARC, subjective age, health, life span development

## Abstract

Combining recent developments in research on personal views on aging (VoA) and a cross-country comparative approach, this study examined awareness of age-related change (AARC) in samples from rural Burkina Faso and Germany. The aims of this study were (1) to examine for an assumed proportional shift in the relationship between gains/losses toward more losses as predicted by life span psychology; (2) to estimate the association between AARC dimensions and subjective age; and (3) to examine the association between health variables and AARC. A cross-sectional method involving a large, representative sample from rural Burkina Faso that included participants aged 40 and older (*N* = 3,028) and a smaller convenience sample of German respondents aged 50 years and older (*N* = 541) were used to address these questions. A proportional shift toward more AARC-losses was more clearly observable in the sample from Burkina Faso as compared to the German reference. In both samples, subjective age was consistently more strongly related to AARC-losses than to AARC-gains. Within the sample from Burkina Faso, differential associations of AARC-gains and AARC-losses to health variables could be shown. In conclusion, the findings support key tenets of life span psychology including that age-related gains occur even late in life and that a shift toward more losses occurs with increasing age. Also, feeling subjectively younger may indeed be more strongly guided by lowered negative aging experiences than by increased positive ones.

## Introduction

Research on subjective views on aging (VoA) has to a large degree made use of constructs such as subjective age or the attitudes toward own aging scale [refer to e.g., (1, 2)]. These measures may be considered reductionistic in terms of narrowing down the subjective experience of aging to one dimension, yet they have gained considerable attention due to their repeatedly shown associations with key developmental and health outcomes, including depression, physical functioning, and even mortality (3–6).

Despite the potential for respective studies to stimulate research on VoA, critique on previous research has been raised due to two major issues: for one, by employing simplified unidimensional measures of VoA, the quintessential multidimensionality of how aging can be perceived and reflected upon is not sufficiently depicted (7, 8). Prominent psychological theories on life span development have, for instance, emphasized that the aging process is marked by both perceived gains as well as losses in multiple domains (9, 10). Addressing this issue has brought forward the concept of “awareness of age-related change” [AARC; (7)]. Essentially, AARC aims to address “(…) all those experiences that make a person aware that his or her behavior, level of performance, or ways of experiencing his or her life have changed as a consequence of having grown older (i.e., increased chronological age)” (7). Looking at previous research on VoA, most older people seem to feel younger than they actually are (2, 11). In contrast, examining AARC-gains and AARC-losses has the potential to inform more specifically about what is behind a reported felt age and may lead to necessary differentiations. For instance, in adults aged 40 years and older, AARC-losses were consistently found to predict subjective age, but also well-being and health, whereas AARC-gains did clearly less so (12, 13).

A second issue that is frequently encountered in research on VoA is that it has, to a considerable degree, relied only on Western samples (11, 14). Aging researchers have ascribed an exceptionally strong role to cultural impacts, for instance, arguing that socioemotional aging in relationships, cognition, and even personality may not manifest in the same way across cultures (15). If cross-cultural research is available, it typically follows a unidimensional VoA approach (14, 16). Hence, using a multidimensional approach to VoA, such as AARC in a cross-cultural approach, may come with the potential to better understand what aging means in a specific culture. For instance, it may shed light on whether AARC-gains at higher ages may currently exist only in some cultures, such as in Western and Asian countries (11, 15), but eventually not in others, such as in African countries. Quantitative evidence on this matter has so far remained scarce. However, a survey of gerontology scholars emphasizes the distinct strengths of older people in the sub-Saharan African context, suggesting that aging as a gainful experience may indeed be an important facet of aging in Africa (17). Particularly, few cross-cultural investigations on the topic of VoA exist from African cultures (11, 18); to date, and to the best of the authors' knowledge, no examination of a multidimensionally framed concept of VoA from an African country's perspective as compared with a Western country's perspective currently exists. We address this gap by relying on the AARC construct and its gains/loss differentiation, as well as by including samples from rural Burkina Faso, Africa, and Germany.

### Life span development and AARC

Of note, life span developmental theories have touched on both of the aforementioned issues. Baltes (9) and also Baltes et al. (10) summarized a family of perspectives that together specify a coherent meta-theoretical view on the nature of development throughout the life course. These include (1) the principle that development should be seen as an interplay of gains and losses across the *full* life span, (2) the principle of multidimensionality and multidirectionality of developmental change, and (3) the unavoidable historical but also cultural embeddedness of developmental processes.

Translating these principles to the subjective interpretation of one's development and getting older as echoed in the AARC construct, the proposed two-factor structure (AARC-gains and AARC-losses) across a range of domains not only mirrors life span psychology's concept of human development but also found initial empirical support in older adults from Germany and the United States (12, 19, 20). Sabatini et al. (21) examined the construct validity of the AARC 10-item short form in a large cohort of participants from the United Kingdom aged 50 years and above and also found support for the two-factor structure of the AARC. Both Kaspar et al. (20) and Sabatini et al. (21) also found support for the age-invariance of both AARC-gain and AARC-loss in their samples. However, whether these results in support of life span developmental principles generalize to non-Western samples with profound differences to Western cultures [Burkina Faso ranks among the lowest countries in development worldwide (22, 23)] remains an open question with importance for the AARC construct and life span developmental psychology at large.

### Health and AARC

As mentioned above, numerous studies have tied VoA to a range of health outcomes, although so far mostly unidimensional measures have been employed [see, e.g., reviews by (3, 4, 24, 25)]. Here, the AARC construct again provides an opportunity for more differentiation in the research field. Early evidence seemed to suggest that AARC-losses play a stronger role in health outcomes than AARC-gains (13). However, a recent study in a large sample of older adults aged 80 years and above has reported that AARC-gains and AARC-losses are independently associated with expected survival times, where AARC-gains appear to function as a protective factor and AARC-losses appear to function as a risk factor, and both showed rather substantial effect sizes (26). Based on their findings on a latent class analysis of AARC-gains and AARC-losses, Sabatini, Ukoumunne (27) not only reported that the combination of high AARC-gains and low AARC-losses was the most common one, making up 45% of the sample from the UK-based PROTECT study, but also that this group had reported much better health across a range of variables than, for instance, participants with many AARC-gains including many AARC-losses at the same time. However, whether such an AARC-health link can also be established in a non-Western sample (in this case, a rural Burkina Faso sample) has not previously been tested.

## Research goals

Building on considerations in life span psychology and extending our previous research (16), we address the following research goals:

First, we examine the assumed proportional shift in the relationship between gains and losses that should take place in older age according to theoretical considerations in life span psychology (9). Given the scarce existing research on cross-cultural differences in VoA at large, it is of special interest whether such a pattern can be identified in a non-Western sample from rural Burkina Faso, given that life span psychology takes a meta-theoretical position aiming to capture essential components of human development that may underlie partial, but not full, cultural framing. As a prerequisite, we also expect to replicate the relatively age-invariant AARC-gain/loss structure in the rural Burkina Faso sample based on confirmatory factor analytical testing [see, e.g., (21)]. Given the uncertainties noted in previous research (18), it is also of great interest to determine whether considerable AARC-gains, hence a positive view on aging, can be observed in rural Burkina Faso, and how this may differ from previously assessed Western samples.

Second, we aim to estimate the relationship between gain/loss in AARC dimensions and subjective age. Essentially, this aims to elucidate what people from rural Burkina Faso might associate with their subjective evaluation of age, and it therefore also targets the validity of the construct across cultures. Based on previous research, we expect AARC-loss to be associated with an older and AARC-gain to be associated with a younger subjective age, though with a comparatively stronger role in terms of the explained variance of AARC-loss (13).

Third, we also examine associations between AARC and several general health parameters in the sample from rural Burkina Faso. This is done with the aim to test whether already established inter-relations among VoA and health in previous research with Western samples also hold in a sample from a markedly different culture and a quite different health context (28).

## Method

### Design and samples

This study presents cross-sectional data and draws from two distinct samples: the main sample from rural Burkina Faso, where data stems primarily from an assessor-based self-report approach, and a German convenience sample, which draws from an online study in which participants answered self-report questionnaires. The two studies had a different primary focus but included measures on VoA and other relevant variables for further use.

The main sample [also described in (16)] was recruited in the district of Nouna, Burkina Faso. The study from which these data originate was conducted within the district of the Centre for Research on Health in Nouna (CRSN) health and demographic surveillance system (HDSS). It contains 58 villages as well as the Nouna town, located in north-western Burkina Faso. The original CRSN Heidelberg Aging Study (CHAS) was conducted between May and July 2018 and aimed to assess the health status of older adults (40 years and above) in the area, including, for instance, cardiovascular risks and cognitive functioning [see (22, 29)]. CHAS sampled 3,998 of approximately 18,000 age eligible HDSS residents from the 2015 HDSS census in two steps. In the first step, in villages with fewer than 50 eligible members, everyone was included. In the second step, everywhere else, a random selection of households containing age-eligible individuals was drawn, and one age-eligible person per selected household was included. About 3,033 individuals provided written informed consent and were included (76% response rate). Clinical measures were assessed by certified research personnel. Interviews were conducted in the participants' preferred language, predominantly (86%) Dioula. Participants were on average 54 years old (*SD* = 11) and with 50% male and 50% female participants, sex was balanced in the sample. A total of 84% of participants from Burkina Faso reported having no formal schooling. Further descriptive data on the sample can be found in Supplementary Table 1.

The German reference sample stems from an online study that was conducted using a database of participants from the infrastructure of the Network Aging Research, Heidelberg University. Potential participants aged 50 years and older were recruited using a mailing list for older adults interested in contributing to aging research. Between July and September 2020, online questionnaires were completed by 541 participants. In contrast to the sample from rural Burkina Faso, the majority of participants (50%) included were from urban areas (i.e., cities with more than 100,000 inhabitants). Participants were on average 69 years old (*SD* = 8), with 57% women and 43% men. About 95% of the German participants had a formal education of 9 years or more. For further descriptive data on the German sample, see Supplementary Table 2.

### Measures: AARC

Awareness of age-related change was measured using the 10-item AARC short form (20). Age-related gains and losses are rated on a five-level Likert scale (1: “not at all” to 5: “very much”) across five life domains: health and physical functioning (PHYS), cognitive functioning (COG), interpersonal relations (INT), social-cognitive and social-emotional functioning (SCSE), and lifestyle and engagement (LIFE), each represented by one item.

All items begin with the phrase “With my increasing age, I realize that (…).” Example items include “(…) I appreciate relationships and people much more” and “(…) I feel more dependent on the help of others” for age-related gains and losses in INT. In the PHYS domain, age-related gains and losses are rated with the items “(…) I pay more attention to my health” for gains, and “(…) I have less energy” for losses. LIFE domain items are “(…) I have more freedom to live my days the way I want” for gains, and “(…) I have to limit my activities” for losses (20). In the sample from Burkina Faso, Cronbach's α amounted to 0.76 (95% CI: 0.74–0.77) for AARC-losses, but more moderate 0.67 (95% CI: 0.65–0.69) for AARC-gains. In the German sample, it was at α = 0.79 (95% CI: 0.76–0.82) for AARC-losses and at α = 0.55 (95% CI: 0.49–0.61) for AARC-gains.

### Measures: Subjective age

Subjective age was measured using the established single item format (“How old do you feel most of the time?”) to which participants responded with their felt age in a numeric format (2, 30). The variable was then converted into a proportional discrepancy score $Subjective\;Age=\left(\frac{Felt\;Age\;-\;Chron.\;Age}{Chron.\;Age}\right)$ by using the participants' chronological age. This score informs about how much older or younger than their chronological age a participant feels in percent. For instance, a score of −0.20 means that they feel 20% younger than their chronological age (2). In some instances, the unrestricted response format can produce unrealistic outliers that may heavily influence linear models. To prevent this, we set responses greater than ±3 *SD*s from the mean to missing.

### Measures: Health variables

Note that suitable health variables were only available for the main sample from rural Burkina Faso, Africa.

#### Functional health

As an approximation for functional health at large in an older population, walking speed was measured. It was defined as the time a participant took to walk 4 m and back at their regular pace (31). The procedure was conducted twice, with the faster of the two walks being counted as the result. Scores were adjusted for gender and height. Walking speed as a measure of functional mobility is predictive of future health outcomes (32, 33).

#### Affective health/depression

The nine-item Patient Health Questionnaire (PHQ-9) was used as an operationalization of depression [see (34)]. In this questionnaire, participants report the extent to which they have experienced symptoms indicating depression on a 4-point Likert scale (from 0 = “not at all” to 3 = “nearly every day”). The instrument has shown its capabilities in assessing depression in an older population and has previously found application in an East African sample (35, 36). Cronbach's α for the scale was at 0.80 (95% CI: 0.79–0.81).

#### Cognitive health

The Community Screening Instrument for Dementia (CSI-D) was used to assess cognitive health. This instrument was specifically developed for the purpose of cognitive screening in cross-cultural research (37). It includes tasks focusing on orientation and episodic memory, which have also shown utility in African samples (38). Higher values indicate better cognition.

#### Quality of life

The WHO Quality of Life (WHOQOL)-Age Scale, which has previously been found to be reliable and valid in the assessment of the quality of life (QoL) across a range of cultures (39), was used to assess participants' QoL. The scale was shortened to eight items that represent key domains for an African population in middle age and old age, including (1) quality of life at large, (2) satisfaction with health, (3) energy in everyday life, (4) activities of daily living, (5) satisfaction with oneself, (6) satisfaction with personal relationships, (7) sufficient money available, and (8) satisfaction with the living place. Answers are provided on a 5-point Likert scale, where higher values indicate better QoL. Cronbach's α in our sample was 0.80 (95% CI: 0.79–0.81).

### Statistical analysis

#### Research question 1

In preparing our analyses, we examine the AARC construct in confirmatory factor analysis for the fit of the proposed two factor model (AARC-gains and AARC-losses) in both the sample from Burkina Faso and the German sample (40). As the sample from Burkina Faso has a sufficiently large size, we also test for the constructs' age-invariance in the sample, splitting it into participants above and below 60 years.

Addressing the first major research question, we then examine the proportion of total perceived gains/losses depending on the participants' age group. For this, we show the empirical distribution of AARC-gains/AARC-losses and the estimated means with standard deviations. On the x-axis, participants' chronological age grouped into bins spanning 10 years each is set, and on the y-axis, the calculated proportion of the total AARC-gains divided by the total AARC-losses is displayed. Note that in order to make the scale for this proportion symmetrical, the data shown on the y-axis is log10-transformed. This procedure is applied to both the data from Burkina Faso as well as the German reference data.

#### Research question 2

For the second research question, linear regression is used to estimate the association of subjective age to AARC dimensions (gains and loss), also taking potential demographic confounders (age and sex) into account. Using standardized beta coefficients, the individual contribution of AARC-gains and AARC-losses in explaining subjective age are then compared within the sample from rural Burkina Faso and the German reference sample.

#### Research question 3

To address the third research question, two sets of hierarchical linear regressions were conducted with AARC-gains and AARC-losses as outcomes, respectively. Building on our previous modeling strategies (16), a block wise procedure of inserting predictors was used. The first block included demographic variables (age, sex, household size, and education); the second block included functional health as measured by walking speed; the third block included affective health (PHQ-9) together with cognitive (CSI-D) health; and the final block included participants' quality of life (WHOQOL).

#### Missing data treatment

In the dataset from Burkina Faso, after excluding five participants without basic assessment (i.e., missing value for gender), the total sample amounted to *N* = 3,028 observations. The remaining missing data amounted to ~1% across all relevant cells. Missing data in the German reference sample was similarly rare for the relevant variables.

For creating the descriptive figures, pairwise deletion was used. In modeling approaches, available data were also used, but the results were examined for robustness using multiple imputations (50 imputation datasets).

All statistical analyses were conducted using R (version 3.6.1).

## Results

### Measurement invariance across countries and age groups

First, we examined the properties of the AARC construct using confirmatory factor analyses (40). The proposed two-factor structure (AARC-gains and AARC-losses) seemed to fit the data from rural Burkina Faso reasonably well, and for the German convenience sample, the fit can be seen as acceptable (see Supplementary Table 3). Furthermore, the two-factor structure appeared to be relatively age invariant in the sample from rural Burkina Faso (tested by splitting the sample into participants above and below 60 years of age; see Supplementary Table 4). Zero-order correlations among AARC items, subjective age, and demographic variables are reported in Supplementary Table 5. In conclusion, these findings support the fact that cross-country and cross-age comparative analyses seem possible.

### Gains/losses across chronological age

**Figure 1:**
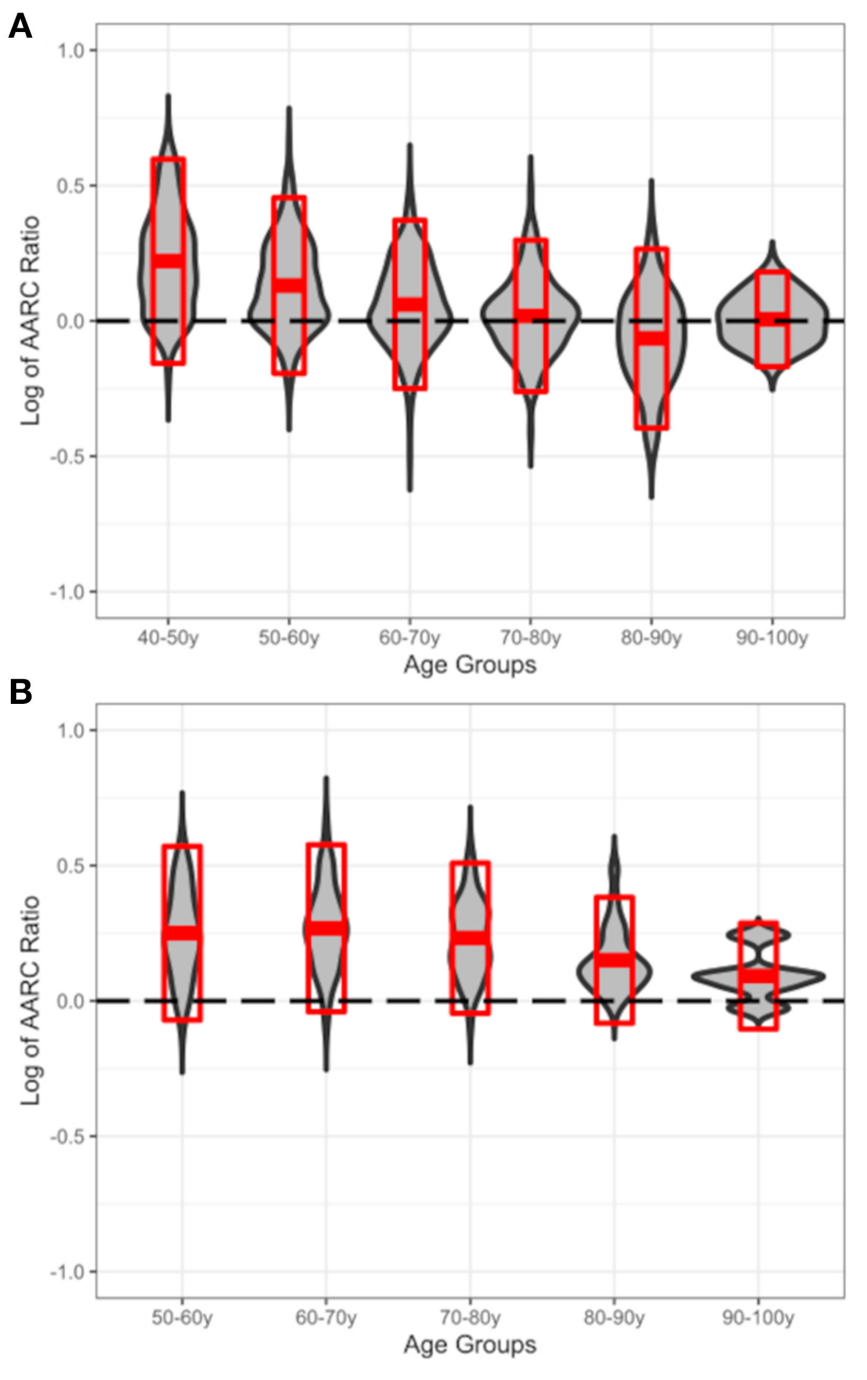
Displayed is the ratio of AARC-gains/AARC-losses (y-axis; log10-transformed for symmetry of the scale) across chronological age groups (x-axis). **(A)** The sample from rural Burkina Faso and **(B)** the German reference sample. The gray violin plot shows the distribution for gains/losses in the respective age groups; superimposed red crossbars display the area of *Mean* ± 1 *SD* within those groups. The dashed reference line indicates a balance between gains and losses (i.e., log of 1:1 ratio), and higher values indicate more gains than losses and lower values *vice versa*.

Figure 1 shows the proportion of AARC-gains/AARC-losses across the age groups; Figure 1A depicts the sample from rural Burkina Faso and Figure 1B depicts the German convenience sample as a reference. Inspecting Figure 1A, which depicts participants from Burkina Faso, a slight downward trend from more gain-related perceptions toward more loss-related perceptions of the aging process with increasing age can be seen. When looking at the German reference in Figure 1B, a similar pattern appears to be observable, although the trend toward more loss-related perceptions of the aging process appears to be weaker and to happen later in life.

### AARC and subjective age

Table 1 shows the results for the association between subjective age and AARC-gains as well as AARC-losses for both, the main sample from Burkina Faso and the German convenience sample. In both samples, among all variables considered (age, sex, AARC-gains, and AARC-losses) for predicting subjective age, AARC-losses had by far the largest effect size, with more AARC-losses being associated with older subjective age. However, it should be emphasized that while AARC-losses were consistently the strongest variable, the overall amount of explained variance was relatively small in both samples, specifically at about 4% for Burkina Faso and somewhat higher at about 10% for Germany.

**Table 1:** Linear regression for the relationship between subjective age (outcome) and AARC-gains as well as AARC-losses controlled for age and sex in the samples from Burkina Faso and Germany.

	**Burkina Faso (*****N*** = **3,028)**		**Germany (*****N*** = **541)**	

	β	**(95% CI)**	β	**(95% CI)**
Age	−0.06^**^	(−0.10, −0.02)	−0.09^*^	(−0.17, −0.00)
Sex	0.04^*^	(0.01, 0.08)	−0.01	(−0.09, 0.08)
AARC-gains	−0.04^*^	(−0.08, −0.01)	−0.07	(−0.16, 0.01)
AARC-losses	0.21^***^	(0.17, 0.25)	0.34^***^	(0.25, 0.42)
*R* ^2^	0.04^***^		0.10^***^	

β are standardized regression coefficients.

^*^p < 0.05; ^**^p < 0.01; ^***^p < 0.001.

### AARC and health

Results on the association between the two AARC dimensions (gains and losses) and health for the sample from rural Burkina Faso are presented in Tables 2, 3, respectively. Looking at AARC-gains, in the first block, lower age, male sex, and higher education in terms of years of schooling were all associated with more AARC-gains. Although sex appeared to be the relatively strongest predictor of these demographic variables, the overall amount of explained variance in the first block only amounted to about 3%. When adding functional health as measured by participants' walking speed in the second block, a small association was found between higher walking speed and more AARC-gains; however, this association was very small and did not noticeably change the overall amount of explained variance. By adding affective (PHQ-9) and cognitive (CSI-D) health in the next step, only a small association of better cognitive health toward more AARC-gains could be shown, and the overall amount of explained variance only increased by about 1 percentage point to a total of 4%. In the fourth and final step, QoL was added to the model. Relatively to the other variables, QoL became the strongest predictor in the model by adding about 3% of explained variance, with higher QoL being associated with more AARC-gains. Yet, it should be noted that all variables of the fourth block together only explained about 7% of the total variance in AARC-gains.

**Table 2:** Hierarchical regression for the association of health variables to AARC-gains as an outcome (Burkina Faso sample; *N* = 3,028).

**Predictor**	**Model 1: Demographics**		**Model 2: Functional health**		**Model 3: Affective and cognitive health**		**Model 4: Quality of life**	

	β	**(95% CI)**	β	**(95% CI)**	β	**(95% CI)**	β	**(95% CI)**
Age	−0.04^*^	(−0.08, −0.00)	−0.02	(−0.06, 0.02)	0.00	(−0.04, 0.04)	0.04	(−0.00, 0.08)
Sex (male ref.)	−0.13^***^	(−0.16, −0.09)	−0.12^***^	(−0.16, −0.08)	−0.11^***^	(−0.15, −0.07)	−0.10^***^	(−0.14, −0.07)
Household size	−0.00	(−0.04, 0.03)	−0.00	(−0.04, 0.03)	−0.01	(−0.05, 0.03)	−0.03	(−0.06, 0.01)
Education	0.08^***^	(0.04, 0.12)	0.07^***^	(0.03, 0.11)	0.06^**^	(0.03, 0.10)	0.06^**^	(0.02, 0.10)
Walking speed			0.05^*^	(0.01, 0.09)	0.04	(−0.00, 0.08)	0.02	(−0.02, 0.06)
PHQ-9					−0.02	(−0.06, 0.02)	0.05^*^	(0.01, 0.09)
CSI-D					0.08^***^	(0.04, 0.12)	0.06^**^	(0.02, 0.09)
WHOQOL							0.21^***^	(0.17, 0.25)
*R* ^2^	0.03		0.03		0.04		0.07	

β are standardized regression coefficients.

^*^p < 0.05; ^**^p < 0.01; ^***^p < 0.001.

**Table 3:** Hierarchical regression for the association of health variables to AARC-losses as an outcome (Burkina Faso sample; *N* = 3,028).

**Predictor**	**Model 1: Demographics**		**Model 2: Functional health**		**Model 3: Affective and cognitive health**		**Model 4: Quality of life**	

	β	**(95% CI)**	β	**(95% CI)**	β	**(95% CI)**	β	**(95% CI)**
Age	0.46^***^	(0.43, 0.49)	0.44^***^	(0.41, 0.47)	0.34^***^	(0.31, 0.38)	0.32^***^	(0.29, 0.35)
Sex (male ref.)	0.14^***^	(0.11, 0.17)	0.13^***^	(0.09, 0.16)	0.09^***^	(0.06, 0.12)	0.08^***^	(0.05, 0.11)
Household size	−0.05^**^	(−0.08, −0.02)	−0.04^**^	(−0.08, −0.01)	−0.03	(−0.06, 0.00)	−0.02	(−0.05, 0.01)
Education	−0.03	(−0.06, 0.00)	−0.03	(−0.06, 0.01)	−0.01	(−0.04, 0.02)	−0.01	(−0.04, 0.02)
Walking speed			−0.03	(−0.06, 0.01)	0.01	(−0.03, 0.04)	0.02	(−0.02, 0.05)
PHQ-9					0.25^***^	(0.22, 0.28)	0.21^***^	(0.17, 0.24)
CSI-D					−0.16^***^	(−0.19, −0.13)	−0.15^***^	(−0.18, −0.11)
WHOQOL							−0.13^***^	(−0.16, −0.09)
*R* ^2^	0.25		0.25		0.33		0.34	

β are standardized regression coefficients.

^**^p < 0.01; ^***^p < 0.001.

Results for AARC-losses, which are presented in Table 3, are visibly different. Starting out with the first model, including demographic variables (older chronological age, female sex, and smaller household size), were related to more AARC-losses. Chronological age was the strongest predictor, and the demographic variables alone already explained roughly 25% of the variance in AARC-losses. Entering functional health in the next block did not notably change model performance. In the third model, an association between less affective and less cognitive health to more AARC-losses could be shown. Besides chronological age, affective health ranked as the most influential variable in the model, followed by cognitive health. Entering these variables also increased the overall amount of explained variance by 8 percentage points to a total of 33%. In the final step, adding QoL had a little further effect on model performance. While lower QoL was shown to be associated with more AARC-losses, this association was not particularly strong compared to the other variables in the model and only added one percentage point to the overall amount of explained variance (full model: 34%).

In summary, differential associations of demographic and health variables with AARC-gains and AARC-losses could be shown, although some similarities (cognitive health) existed. Demographic and health variables appear to be much better suited to explain AARC-losses than AARC-gains.

## Discussion

Previous research on VoA has emphasized the need for better consideration of the multidimensionality and multidirectionality of VoA and the necessity of more cross-cultural examinations. In particular, data able to provide insights about VoA from the African cultural context is rare (11, 18, 41). This study presents, to the best of the authors' knowledge for the first time, data on AARC in an African sample from rural Burkina Faso. Furthermore, and for selected research questions, this study also made an initial attempt at a cross-country comparative approach by referencing results from rural Burkina Faso to a convenience sample from Germany. We are well aware that a convenience sample comes with limitations, and therefore the interpretation of all comparative findings deserves caution. On the contrary, having such a comparison option available as we did can be seen as a first step in learning about possible differences in VoA in cultures quite different from each other.

First, we examined the properties of the two-factor structure (AARC-gains and AARC-losses) of the AARC instrument (20). Early on, life span psychology considered the interplay of gains and losses, of growth and decline, as indicative of the aging process (9). As Smith (42) pointed out, despite their acceptance as a metalevel developmental concept, these ideas have rarely been examined empirically. Our findings are in support of the proposed two-factor structure derived from life-span psychological considerations (9, 10). This is of special importance given the meta-theoretical perspective that life span developmental psychology aims for, which should remain valid across a range of different cultural settings.

Furthermore, going into more detail about how gains and losses are perceived across the life course, we also investigated the proportion of overall AARC-gains and AARC-losses across age groups. Baltes (9) specifically predicted that a proportional shift from a more gain-dominated to a more loss-dominated interplay of gains and losses would take place in later life. Such a pattern was rather distinctly observable in the sample from rural Burkina Faso, supporting these fundamental considerations of life span psychology in a non-Western sample. Within the German convenience sample, a similar pattern was also observable albeit with a less pronounced and later shift toward AARC-losses, as was observable in the Burkina Faso data. Explanations at different levels could help shed light on these findings.

A first explanation might be that degrees of freedom for developmental regulation might be larger in Western samples. The model of selective optimization with compensation (SOC) argues that being selective and using compensatory means may buffer the experience of loss in later life and indeed supports ongoing developmental optimization at least to some extent [see, e.g., (43, 44)]. Qualitative research by Nimrod (45) points, for example, to the utility of information and communication technology in SOC processes, and the use of such technologies is increasing even in very old age in the Western sphere (46). In a developing country, such as Burkina Faso, thresholds to the use of information and communication technology, or more generally, issues in the healthcare system, may, as a consequence, offer fewer possibilities for successfully enacting SOC strategies in later life (47).

Second, a rather clear cohort flow has been observed in Western countries toward increasing health, functioning, and decreasing loneliness and external control beliefs (48), which might have happened to a much lesser extent in Burkina Faso, although no detailed data on such cohort change is available in this country. The positive cohort change in Western countries might coincide with the experience of less AARC-loss and more AARC-gain from a historical perspective.

Third, given that tendencies toward more AARC-losses were also reported in a Western sample [data from the United Kingdom; see (21)], an alternative explanation at the level of methodology should also be considered for why the German sample did not more clearly exert such a pattern. Of note, as opposed to the sample from rural Burkina Faso, our German sample was a convenience sample of most likely in multiple aspects positively selected online survey participants. For instance, German participants in the convenience sample have an above average education level. Such selective participation has the potential to introduce biased results, and instead of drawing definite conclusions regarding the results of the German sample, we see this as a strong opportunity for future research to provide representative Western data on the proportion of AARC gains/losses to allow for more valid comparisons. However, the findings from both samples are very much interpretable in support of an even more fundamental statement of life span psychology, that is, that age-related gains are still experienced even in old age (9, 42, 49).

We further examined the association between subjective age and AARC. In a recent study (16), we noted that the cross-cultural validity of the subjective age construct has not yet been convincingly shown. Indeed, it is a difficult question whether or not feeling older or younger is interpreted similarly in different cultures. The improved but compared to Western countries still lower life expectancy in Burkina Faso (50) means that a different scaling of when “older age” starts is likely to exist. Although older age therefore likely begins earlier in Burkina Faso than in Germany, our result that in both samples AARC-losses were to a similarly strong degree associated to subjective age lends partial support to the idea that this construct is interpreted similarly in different cultures. Our results are also in support of a previous study that has reported that subjective age was more strongly related to AARC-losses than to AARC-gains (13).

Finally, we investigated the association between health variables and AARC-gains as well as AARC-losses in the sample from Burkina Faso. Among the considered variables, QoL showed a relatively strong association with AARC-gains, although overall, demographic and health variables only explained little variances in total. In contrast, when AARC-losses were considered, older chronological age and also depression showed relatively strong associations with more AARC-losses. On the one hand, this supports a previous study in which particularly affective health, such as depressive symptoms, was shown to explain a considerable proportion of variance in negative VoA (5, 51–53). On the other hand, and likewise in accordance with the previously mentioned finding that subjective age was more strongly related to AARC-losses than AARC-gains, the clearly stronger connection of important health variables to AARC-losses substantiates previous studies that have noted that negative VoA may be relatively more important for behavioral outcomes, which are in turn tied to health consequences (4, 13, 54, 55). We interpret this finding with all caution as a first hint that more negative VoA represents more of a health threat than more positive VoA provides a buffer toward better health at a general level, thus independent of culture. The fact that the sex of the participants played a role in both AARC-gains and AARC-losses may, however, be a manifestation of sex-specific challenges when growing older in rural Burkina Faso. Non-governmental organizations have pointed to several challenges that especially women may face in this context, such as loss of social status through widowhood or accusations of witchcraft (56). Future research may benefit from including qualitative methodology to better understand what it means to age in Burkina Faso for both women and men.

### Limitations

Several limitations must be taken into account when interpreting the results of this study. First and foremost, this study presented cross-sectional data and, therefore, by design, neither causal inferences nor conclusions about the directionality of effects can be made. Furthermore, this is an exploratory study that draws in part from convenience sampling and lacks an *a priori* published analysis protocol. While there was missing data in both datasets, the number was considerably low, and the results of the analyses were robust, i.e., available cases and analyses based on multiple imputations were very similar. However, this does not address the issue of possible selection bias in participant recruitment by which the German convenience sample might be affected. Especially the results from the German sample have to be treated with caution as the sample is not representative, and therefore, participants with more positive VoA might have self-selected in participation, as they are assumed to be more active in general. Although we examined the factor structure of the AARC instrument in both samples, this did not guarantee cross-cultural validity. Qualitative research could supplement this by inquiring about the meaning ascribed to perceived gains and losses in different cultures. Finally, all limitations of self-report measures must be considered.

## Conclusion

The findings on AARC-gains and AARC-losses presented in this study largely support fundamental propositions brought forth by life span psychology, implying their meta-theoretical and possibly universal nature. To this end, we observed perceived AARC-gains even in older age and even in our Burkina Faso sample, although with increasing age a shift toward more losses took place. Still, the proportional shift to AARC-losses was clearly stronger in the Burkina Faso sample as compared to the German reference sample. This may be seen as supporting the “classic” two-factor impact of getting older and culture on human development.

## Data availability statement

The data analyzed in this study is subject to the following licenses/restrictions: Data was used under license. Requests to access these datasets should be directed to TB (BF data) or AnnS (Ger data).

## Ethics statement

The studies involving human participants were reviewed and approved by Ethics Committee of the University of Heidelberg's Faculty of Medicine, Comité national d'éthique pour la recherche en santé in Ouagadougou, and CRSN Comité d'éthique institutionnelle. The patients/participants provided their written informed consent to participate in this study.

## Author contributions

ASchö: conceptualization, analysis, and writing—original draft. ASchl: data administration and writing—review and editing. H-WW and TB: conceptualization, project supervision, and writing—review and editing. All authors contributed to the article and approved the submitted version.
