# Supplementary material for: Awareness of age-related change in very different cultural-political contexts: A cross-cultural examination of aging in Burkina Faso and Germany
Source: Front Psychiatry. 2023 Jan 20;13:928564. doi: 10.3389/fpsyt.2022.928564 (PMC9894898; doi:10.3389/fpsyt.2022.928564)
Supplement: Supplementary file 1 [file Data_Sheet_1.PDF]

Supplementary Table 1

Descriptive Statistics for the Sample from Rural Burkina Faso (N=3028).

Variable	Mean	SD	Min	Max	Median	Q1	Q3	Missings
Age	54.31	11	40	103	52	45	62	2
Education (years)	1.01	2.86	0	38	0	0	0	0
Walking speed	0.97	0.26	0.12	2.06	0.97	0.79	1.05	98
PHQ-9 score	4.38	3.57	0	23	4	2	7	0
CSI-D score	8.41	1.1	2	9	9	8	9	0
WHOQOL (norm.)	56.01	14.32	9.38	96.88	59.38	46.88	65.62	0
Subjective age (prop. score)	-0.03	0.13	-0.56	0.46	0	-0.09	0.02	74
AARC Int+	3.48	1.18	1	5	4	2	4	0
AARC Phys+	3.63	1.16	1	5	4	3	5	0
AARC Cog-	2.22	1.1	1	5	2	1	3	0
AARC Cog+	3.19	1.1	1	5	3	2	4	0
AARC Scse+	3.57	1.08	1	5	4	3	4	0
AARC Life-	3.01	1.27	1	5	3	2	4	0
AARC Phys-	2.84	1.13	1	5	3	2	4	0
AARC Int-	2.3	1.26	1	5	2	1	3	0
AARC Life+	2.59	1.19	1	5	2	2	3	0
AARC Scse-	2.18	1.05	1	5	2	1	3	0

Note: PHQ-9 = Patient Health Questionnaire-9; CSI-D= Community Screening Instrument for Dementia; WHOQOL= WHO Quality of Life scale; AARC Domains: INT = Interpersonal Relations; PHYS = Health and Physical Functioning; COG = Cognitive Functioning; SCSE = Social-Cognitive and Social-Emotional Functioning; LIFE = Lifestyle and Engagement. In AARC Items "+" denotes items indicating gains & "-" denotes items indicating losses.

Supplementary Table 2

Descriptive Data for the German Convenience Sample (N=541).

Variable	Mean	SD	Min	Max	Median	Q1	Q3	Missings
Age	68.85	8.2	50	94	69	64	74	0
Subjective age (prop. score)	-0.12	0.12	-0.85	0.35	-0.11	-0.19	-0.03	10
AARC Int+	3.56	1.02	1	5	4	3	4	2
AARC Cog-	3.31	1	1	5	3	3	4	1
AARC Phys+	2.23	0.8	1	5	2	2	3	1
AARC Scse+	3.85	0.86	1	5	4	3	4	1
AARC Phys-	3.9	0.86	1	5	4	4	4	1
AARC Cog+	2.36	0.95	1	5	2	2	3	1
AARC Life-	2.62	0.93	1	5	2.5	2	3	1
AARC Int-	1.86	0.92	1	5	2	1	2	1
AARC Life+	3.89	1.14	1	5	4	3	5	1
AARC Scse-	1.93	0.95	1	5	2	1	3	1

Note: AARC Domains: INT = Interpersonal Relations; PHYS = Health and Physical Functioning; COG = Cognitive Functioning; SCSE = Social-Cognitive and Social-Emotional Functioning; LIFE = Lifestyle and Engagement. In AARC Items “+” denotes items indicating gains & “-“ denotes items indicating losses.

Supplementary Table 3a

Indices of Fit for the Confirmatory Factor Analyses Examining the two Factor Structure of the AARC Construct in the Sample from Burkina Faso as well as the German Sample.

Analysis set	CFI	SRMSR	RMSEA [90% CI]
Burkina Faso	0.912	0.046	0.072 [.067; .077]
Germany	0.887	0.081	0.080 [.067; .094]

Note: CFI = Comparative Fit Index; SRMSR= Standardized Root Mean Square Residual; RMSEA= Root Mean Square Error of Approximation.

Supplementary Table 3b

Factor Loadings of the AARC-10-SF Items (with 95% CIs) on the Latent Variables for the two Factor Structure (Gains, Losses). Samples from Rural Burkina Faso and Germany.

Burkina Faso Sample						
	AARC-gains			AARC-losses		
INT+	.62	[.59;	.65]			
PHYS+	.59	[.56;	.62]			
COG+	.65	[.62;	.68]			
SCSE+	.65	[.62;	.68]			
LIFE+	.21	[.17;	.25]			
COG-				.51	[.48;	.54]
LIFE-				.72	[.70;	.75]
PHYS-				.73	[.70;	.75]
INT-				.60	[.57;	.63]
SCSE-				.54	[.51;	.57]
German Sample						
	AARC-gains			AARC-losses		
INT+	.41	[.31;	.51]			
PHYS+	.34	[.24;	.44]			
COG+	.56	[.47;	.66]			
SCSE+	.67	[.57;	.77]			
LIFE+	.31	[.21;	.41]			
COG-				.50	[.43;	.57]
LIFE-				.74	[.69;	.79]
PHYS-				.83	[.78;	.87]
INT-				.60	[.53;	.66]
SCSE-				.60	[.53;	.66]

Note: AARC Domains: INT = Interpersonal Relations; PHYS = Health and Physical Functioning; COG = Cognitive Functioning; SCSE = Social-Cognitive and Social-Emotional Functioning; LIFE = Lifestyle and Engagement. In AARC Items “+” denotes items indicating gains & “-“ denotes items indicating losses.

Supplementary Table 4

Indices of Fit for the Confirmatory Factor Analyses Examining Age-invariance of the AARC Construct in the Sample from Rural Burkina Faso.

Analysis	CFI	SRMSR	RMSEA [90% CI]
Configural invariance	0.895	0.046	0.073 [.068; .078]
Weak invariance	0.894	0.047	0.070 [.065; .075]
Strong invariance	0.890	0.049	0.067 [.063; .072]

Note: Age groups were created by splitting the sample into participants below and above 60 years of age. CFI = Comparative Fit Index; SRMSR= Standardized Root Mean Square Residual; RMSEA= Root Mean Square Error of Approximation.

Configural invariance= imposition of the same factor structure across groups; weak invariance= constraining equal factor loadings across groups; strong invariance= constraining factor loadings and intercepts to be equal across groups.

Supplementary Table 5

Zero-Order Correlations of Subjective Age, AARC Items, and Demographic Variables. Below the Diagonal are the Values for the Sample from Rural Burkina Faso, above the Diagonal are Values from the German Sample.

	1.	2.	3.	4.	5.	6.	7.	8.	9.	10.	11.	12.	13.
1. Age		-0.12	-0.01	0.00	-0.02	0.13	-0.09	-0.13	0.23	0.16	0.18	0.1	0.07
2. Sex	0.10		-0.02	0.02	-0.11	-0.10	-0.08	0.10	-0.06	-0.06	-0.06	-0.1	-0.02
3. Subj. Age	0.05	0.08		-0.02	-0.01	0.18	-0.06	-0.02	0.25	0.30	0.21	-0.05	0.21
4. AARC Int+	-0.04	-0.08	0.01		0.18	0.21	0.27	0.23	0.09	0.07	0.12	0.11	0.05
5. AARC Phys+	-0.02	-0.07	-0.01	0.44		0.18	0.12	0.24	0.24	0.18	0.18	0.12	0.11
6. AARC Cog-	0.31	0.13	0.11	-0.09	0.02		-0.06	-0.02	0.37	0.37	0.38	-0.05	0.33
7. AARC Cog+	-0.03	-0.09	-0.04	0.38	0.32	-0.01		0.39	0.02	0.02	0.01	0.17	-0.07
8. AARC Scse+	-0.11	-0.10	-0.06	0.34	0.39	-0.10	0.48		0.01	0.08	0.03	0.22	-0.10
9. AARC Life-	0.43	0.16	0.18	-0.06	0.09	0.37	0.03	0.02		0.62	0.43	-0.09	0.42
10. AARC Phys-	0.39	0.14	0.14	-0.03	0.05	0.37	0.01	-0.04	0.56		0.49	-0.10	0.51
11. AARC Int-	0.33	0.15	0.12	-0.05	-0.01	0.28	-0.02	-0.09	0.41	0.42		-0.21	0.34
12. AARC Life+	-0.02	-0.13	-0.06	0.19	0.09	-0.03	0.14	0.10	-0.02	0.04	0.11		-0.15
13. AARC Scse-	0.26	0.12	0.11	-0.04	0.01	0.30	-0.02	-0.06	0.35	0.35	0.45	0.09	

Note: AARC Domains: INT = Interpersonal Relations; PHYS = Health and Physical Functioning; COG = Cognitive Functioning; SCSE = Social-Cognitive and Social-Emotional Functioning; LIFE = Lifestyle and Engagement. In AARC Items “+” denotes items indicating gains & “-“ denotes items indicating losses.
